# Egoistic punishment outcompetes altruistic punishment in the spatial public goods game

**DOI:** 10.1038/s41598-021-85814-1

**Published:** 2021-03-22

**Authors:** Juan Li, Yi Liu, Zhen Wang, Haoxiang Xia

**Affiliations:** 1 School of economics and management, Dalian University of Technology, Dalian, 116024 China; 2 Department of Public Administration, Dalian University of Technology, Dalian, 116024 China; 3 School of Cyberspace, Hangzhou Dianzi University, Hangzhou, 310018 China

**Keywords:** Social evolution, Human behaviour

## Abstract

The evolution of costly punishment is a puzzle due to cooperators’ second-order free-riding. Previous studies have proposed many solutions mainly focused on reducing the punishment cost or punishing second-order free riders directly or indirectly. We attempt to explain this confusion from the perspective of punishment motivation, which is why the punisher is willing to pay the cost. The answer is that the punisher is egoistic. Egoistic punishment aims to protect punishers’ own cooperative benefits shared by the defectors. In such case, egoistic punishers would pay a cost in punishing defectors and retrieve lost payoffs from defectors. Here, we examined the evolution and performance of egoistic punishment and compared it with typical altruistic punishment using classic peer-punishment and pool-punishment modes. Results showed egoistic punishment can evolve and effectively promote cooperation within a large parameter range, whether in a well-mixed or structured population, or through peer-punishment or pool-punishment modes. This result is also robust to different strategy-updating rules. The evolution under the pool-punishment mechanism is more complicated. The influence of parameters is counter-intuitive because of cycle dominance; namely, the cost is the key factor to control the level of cooperation and the fine determines the ratio of the punishers and cooperators. Compared with altruistic punishment, egoistic punishment can promote cooperation in a lower-fine and higher-cost area, especially in the pool punishment mode, and the egoistic punishers have stronger survivability. Egoistic punishers represent the natural fairness in a social system. Results revealed that focusing on individual equity can significantly promote collective cooperation. This study provides another explanation for the evolution of costly punishment.

## Introduction

“Tragedy of the Commons” is a wicked problem in society^1, 2^. The increasing demand for common-pool resources (CPR), such as inshore fishery, forests, and clean water, widely results in resources degradation. Even though cooperative constraints can help maintain sustainable resources exploitation, the self-interested strategy (typically free-riding) frequently induces a temptation to defect rather than cooperate, and leads to resources exhaustion. The uniform solutions for CPR dilemmas are absent, and effective governance arrangements are scenario dependent^3^. Communities rely on the self-organizing governance system in parallel with the government’s external enforcement. The public goods game (PGG) engaged in exploring this conflict between individuals and the common benefit discovered a considerable number of approbatory supportive mechanisms which underpinned the complexity in solving the CPR problem, mainly including direct and indirect reciprocity^4, 5^, group selection^6^, network reciprocity^7^, optional participation^8^, and punishment and reward^9, 10^. Among them, punishment attracts much scholarly attention, in that punishment entails direct and short-term costs in comparison with the other mechanisms, which echoes the critical elements for CPR governance. Punishing the defector is an effective way to incentivizee users to invest in CPR^9, 11^, but there is a new question to be solved: How does the costly punisher evolve due to cooperators’ second-order free-riding?

Scientists proposed many valuable solutions to understand the emergence and evolution of costly punishment. Adding a spatial interaction structure is a well-known solution in which the punisher is spatially separated from the cooperator^12–14^. An intuitive solution is to punish second-order free-riders (i.e., pure cooperators) in the structured population^15^, or before first-order punishment^16^, or execute it by democratic decisions with majority-voting rule^17^. Sharing punishment costs with cooperators^18^ or among the group members^19^ by collective punishment, or declining the total cost as the punisher increases (as defined by coordination punishment)^20^, are also effective solutions. The heterogeneity of punishers, instead of homogenous punishment, plays an important role in explaining the costly punishment, such as punitive preferences, monetary incentives and tacit coordination^21^, the punisher’s cooperativeness^12^, the punisher’s power asymmetry^22^, the leader-based punisher^23^, the probabilistic punisher who punishes the opponent at high probability if the payoffs’ difference is large^24^. In addition, the puzzle can be solved by considering additional factors, such as group selection^25^, reputation^5^, and social learning^26, 27^. Moreover, introducing a loner strategy that voluntarily participates in the contribution game^28^, or adding an opportunity strategy that contributes to the public good only if the punishment institution is established^29^, paves the way to the evolution of punishment. Considering the punishment mechanism of different scenarios indirectly promotes the evolution of altruistic punishment. Conditional punishments that make punish decision depend on^22^ or align proportionally with^30^ the number of unconditional punishers, or only considering the willingness of punishment^31^. There are also antisocial punishment of defectors to punish cooperators^32^, and the selfish punishment of defectors to punish other defectors^33, 34^. Furthermore, social exclusion, the variant of punishment, prevent free-riders share cooperative benefits^35^ is also a solution. Overall, existing research provides effective solutions to directly solve the second-order free-riding problem by reducing or offsetting the punishment cost and reducing the evolutionary disadvantages of the punisher by second-order punishment; or indirectly solving the problem by introducing strategies, factors, and competitions between punishment mechanisms.

It is worth pointing out that Guala^36^ and the associated open-peer commentaries discuss the second-order free riding problem and the strategic nature of costly punishment by explaining the limits of costly punishment and the ways it might overcome those limits. In addition, inspired by human egoism in reality, it is possible to explain the evolutionary puzzle of costly punishment from the perspective of punishment motivation; that is, to first answer why individuals are willing to pay additional costs to punish defectors.

Scientists carried out many valuable studies to investigate the motivation to punish defectors. Altruism attracts much attention. Fehr and Gächter^37^ showed experimentally that cooperators have a widespread willingness to punish defectors, even if they cannot receive any benefits from their punishment activities, reflecting a pure altruistic punishment tendency. The proximate motive is the negative emotions towards defectors^38^, or the egalitarian motives to receive a fair benefit^39^. The neuroscience evidence confirmed the neural basis of altruistic punishment, and psychologically explains motivation as an impulsive behavior^40^, or a behavior to obtain satisfaction^41, 42^. Although altruistic punishment is widespread in laboratory experiments, it is hard to find evidence in the real world^36^. Altruistic punishment may be overstated^43^; however, punishing the defector often brings direct or indirect benefits. The punisher builds a good reputation by punishing the defector so that he can get more help in subsequent games^44^. After being punished, defectors are transformed into cooperators, thereby increasing the group benefits. Reciprocal altruism seems to be a more realistic motivation and resembles egoism as to the motivation for punishing the defector. Rand and Nowak^45^ researched the full set of punishment strategies and indicated punishment is mostly a self-interested tool for protecting oneself against potential competitors. This research shifts the motivation behind punishment from the altruistic to egoistic perspective. Researchers are generally quite cautious with the assumption of the punisher’s motivation and the “egoistic punisher” is seldom spotted. In some literatures, the egoistic punishers’ motives are to avoid altruistic punishers’ sanctions (i.e., behaving selfishly when it comes to public goods contribution whilst punishing other defectors altruistically^33, 34^).

The illustration of egoistic punishers who are broadly defined can be observed from the real world. As Hirshleifer^46^ noted, the distinctive punisher’s behavior, the so-called “bounty hunter,” exploits defectors while being nice to cooperators. In tracing back to ancient Rome, war trophies provided undeniable motives, thus driving Roman soldiers to make sacrifices^47^. Nowadays, courts have recognized punitive damage as a punishment for illegal acts and provide compensation to plaintiffs^48^. Also, as the “endowment effect” states, proposed by Richard Thaler^49^, people’s consideration of “avoiding harm” is greater than their consideration of “seeking profit.” That is, the motivation of individuals who impose punishment on defectors is more likely to protect their potential cooperative benefits from losses than to obtain uncertain rewards. The above insights from social scientists make us believe it is necessary to investigate the performance of egoistic punishers and discern its dominance in enhancing cooperation by conducting comparative studies in between egoistic and altruistic punishment.

Here, we explored the evolution of egoistic punishment in two modes, peer and pool punishment, and compared their performance with classic altruistic punishment. Four types of punishment and corresponding representative punishers appear in Table 1. In egotistic peer-punishment (*EP*), the punisher pays a cost to punish in order to acquire a reward from the fine imposed on defectors, called the Bounty Hunter ($P_B$). In egotistic institutional punishment (*EI*) (we use institutional in the abbreviation instead of pool to distinguish it from peer), the punisher pays dues and acquires a reward that comes from the defector’s fine. We call this kind of punisher Clans ($P_S$). As a comparison, in altruistic peer-punishment (*AP*), the punisher pays a cost as an individual to punish defectors, even if they receive no reward. We call this Robin Hood ($P_R$). In altruistic institutional punishment (*AI*), the punisher pays a tax to build a public punishment institution and impairs the benefit to punish defectors without a commensurate material payoff, dubbed Government-like ($P_G$). Egoistic punishment is as enforceable as altruistic punishment in solving CPR dilemmas. For example, fines will be imposed on those who deforest, and fines will be imposed on fishing boats that still fish during the fishing moratorium. Egoistic punishment motivates individuals to actively discover these uncooperative individuals.

**Table 1:** Four types of punishment.

		Punishment modes	
		Peer	Institutional/pool
Punishers’ motivation	Altruistic	*AP* Robin Hood	*AI* Government
	Egoistic	*EP* Bounty hunter	*EI* Clans

The PGG model implemented this comparative study with a punishment mechanism. Cooperators (*C*) contribute to the common pool, and defectors (*D*) contribute nothing but share the fruits of cooperation. In the punishment stage, some cooperators become punishers ($P_i, (i=B,S,R,G)$) to punish the defector motived by egoism ($i=B,S,$) or altruism ($i=R,G,$). Egoistic punishers have the characteristic of “loss aversion” and punish defectors to retrieve the fruits of cooperation the defectors stole. Specifically, one punisher retrieves some fine ($\beta *r/5$) from each defector to compensate for the lost cooperative payoff (because a defector takes a free payoff of *r*/5 from a cooperator in the group). In general, a defector’s fine ($\beta *r/5*n_{P_i}$) is proportional to the number of egoistic punishers, and the punishment payoff of an egoistic punisher is also proportional to the number of defectors ($\beta *r/5*n_D$). As described, an altruistic punisher punishes the defector for public order rather than for their own personal payoff and fines ($\beta$) reduce the punished defector’s payoff. Meanwhile, the punishment cost is proportional to the number of defectors ($\alpha *n_D$) in peer punishment; while in pool punishment, the punishment cost is a fixed value ($\alpha$). See the methods section for a detailed description of the model and experiment.

Variable $\beta$ stands for the defector’s fine and its value is $0<\beta \le 1$, indicating the punishment tolerance. $\beta =1$ means egoistic punishers will take back all cooperative payoffs generated by the punisher’s contribution from the defector. Variable $\alpha$ stands for punisher’s cost and its value is $0\le \alpha \le 1$. The parameter *r* is the synergy factor, also called the cooperative coefficient. Researchers found that cooperators manage to survive if $r>3.74$, and crowd out other strategies if $r>5.49$^50^. We chose the synergy factor’s normative values $r (r = 2.0, 3.5, 3.8,$ and 4.0) in our experiment to reveal the possible stable solutions.

In particular, it should be pointed out that the egoistic punishment has no obvious evolutionary advantage than the altruistic punishment, although the egoistic punisher’s return may offset part of the cost through punishment and the altruistic punisher does not have any return because the defector in altruistic punishment is punished much more severely than in egoistic punishment. Specifically, in the peer-punishment mode, a defector loses benefits $\beta *N_{P_R}$ when encountering altruistic punishers ($P_R$), whereas the defector loses benefits $\beta *{r/5}*N_{P_B}$ when encountering egoistic punishers ($P_B$). In the pool-punishment mode, a defector loses benefits $\beta$ when encountering altruistic punishers ($P_G$); whereas the defector loses benefits $\beta *r/5*N_{P_S}$ when encountering egoistic punishers ($P_S$), which is dependent on the number of $N_{P_S}$ in the group. It is difficult to intuitively judge the evolutionary advantage of the egoistic punishment given that the punisher’s and defector’s fitness of egoistic punishment are both higher than that of altruistic punishment, which makes our research meaningful.

Our main results for egoistic punishment in the PGG may be summarized as follows. First, egoistic punishment can evolve and effectively promote cooperation, whether in the peer-punishment or pool-punishment mode. The result is robust under different strategy-update rules. The main difference is that egoistic peer-punishment can quickly eliminate defectors and evolve the population into full cooperation, while egoistic pool-punishment can maintain a certain level of cooperation in areas with lower fines and higher costs and the punishers coexist with other strategies. In addition, the evolution under the pool-punishment mechanism is more complicated. The parameters first take effect and then the three strategies’ cycle dominance changes the evolutionary result. The parameters’ role on the evolution is non-intuitive; namely, the cost is the key factor to control the level of cooperation and the fine determines the ratio of the punishers and cooperators. Then, compared with altruistic punishment, egoistic punishment can promote cooperation in a lower-fine and higher-cost area, especially in the pool-punishment mode. The egoistic punishers have stronger survivability than altruistic punishers, especially in the middle- or higher-cost area. Finally, a moderate fine is the most appropriate for both egoistic and altruistic pool punishments. A high fine is not conducive to the punisher’s survival, which, in turn, leads to the unsustainability and ineffectiveness of the punishment mechanism.

## Results

We first focus on the performance of egoistic punishment in the well-mixed population. As we all know, a peer altruistic punisher cannot survive in a well-mixed population and a pool altruistic punisher can only prevail assuming the additional punishment of pure cooperators^51^. However, egoistic punishers can survive without further complexity, as shown in Figure S1 and S2 in the supplementary material. Pool egoistic punishers prevail as the fine ($\beta$) increases or as the punishment cost ($\alpha$) decreases, and the system consecutively transitions from the pure $\mathrm D$ phase to the $\mathrm D+P_S$ phase, then to the $\mathrm C+D+P_S$ phase. Peer egoistic punishers replace defectors and then are invaded by pure cooperators as $\beta$ increases or as $\alpha$ decreases. Accordingly, the system discontinuously transitions from the pure $\mathrm D$ phase to the pure $\mathrm P_B$ phase, and then consecutively transitions to the $\mathrm P_B+C$ phase. Both in peer and pool punishment, the phase transition boundaries of $(\beta , \alpha )$ move left as the cooperative coefficient (*r*) increases. The difference is that the critical lines are linear in the pool mode, while they are nonlinear in the peer mode.

The performance of egoistic punishment is highlighted in the well-mixed population and the influence of perimeters on the egoistic punishment mechanism seems obvious. Actually, the fitness of egoistic punishers is not only related to $\alpha$ and $\beta$, but also to the number of defectors. The fitness of the defector is also related to the number of punishers. It is difficult to portray and demonstrate the evolution of such complex relationships in a well-mixed homogeneous system. Therefore, studying the evolution and cooperation of complex dynamic process in a structured population is necessary. By introducing a four-neighbor lattice structure, the population is split into heterogeneous interactive groups. Despite its simplicity, the spatial model exhibits really complex behavior in different spatial and time scales.

We are interested in who will be the winner between punishers motivated by egoism and altruism by examining which mechanism can achieve a higher cooperation level and how the introduced punishment strategy performs. We first explored the performance of egoistic punishment proposed in this study and its fundamental mechanism for promoting cooperation. Then we compared the performance of egoism with typical altruism under the peer-punishment and pool-punishment modes from the level of cooperation, the evolutionary equilibrium (EE), and the punisher’s survivability.

### Performances of egoism under peer- and pool-punishment modes

**Figure 1:**
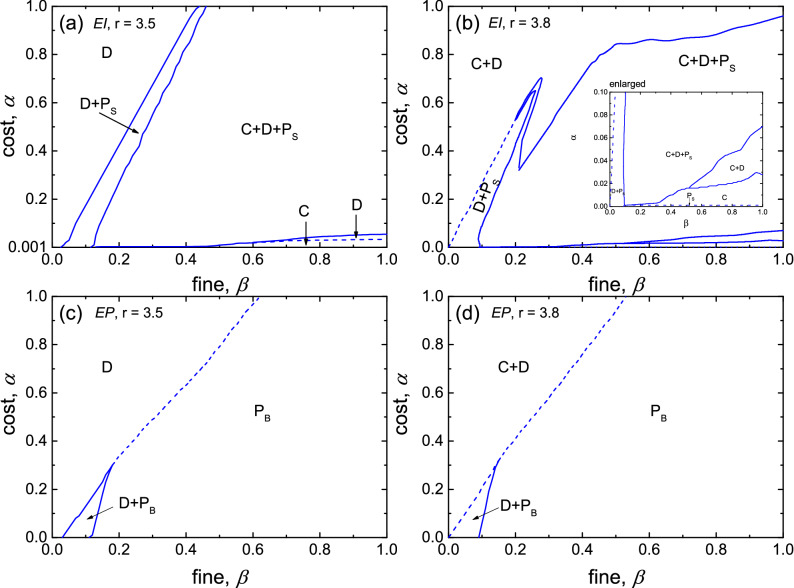
Full fine-cost phase diagrams of egoistic punishment in the structured population. Solid (dash) lines indicate continuous (discontinuous) phase transitions. The results show the solutions are significantly different under different penalty modes. The phase transitions in egoistic pool punishment are more complex.

First, we illustrate the phase transitions of *EI* and *EP* punishment in the full fine-cost areas for $r=3.5$ (the cooperators cannot survive in the absence of egoistic punishment) and 3.8 (the *C* and *D* coexist in the absence of punishment) by systematic Monte Carlo (MC) simulations. In each case we have determined the stationary frequencies of strategies when varying the fine $\beta$ for many fixed values of cost $\alpha$. The transition points and the type of phase transitions are identified from the dataset. The phase boundaries are plotted in the full fine-cost phase diagrams, as shown in Fig. 1.

The phase diagram of *EI* in the structured population for $r=3.5$ (as Fig. 1a shows) is similar to that in the well-mixed population. But due to the spatial structure’s restrictive interaction, the phase boundary value of ($\beta , \alpha$) required for the egoistic punishers’s survival is lower. The phase transitions from the pure D phase to the $\mathrm D+P_S$ phase, then to the $\mathrm C+D+P_S$ phase as $\beta$ increases. When increasing the cost $\alpha$ at a high value of fine ($\beta =0.9$), one can observe three discontinuous transitions from the pure P ($\alpha =0$) to the pure C phase, to the pure D phase and then to the $\mathrm C+D+P_S$ phase. In the D phase, punishers are first eliminated by pure cooperators, and then defectors invade the cooperators and prevail. Below this phase, the defector is eliminated first, and above this phase, the three strategies coexist under the cyclic dominance.

The phase diagram changes a lot when $r=3.8$, as Fig. 1b illustrates. The $\mathrm D+P_S$ phase is surround by the $\mathrm C+D+P_S$ phase when the value of ($\beta , \alpha$) is higher, which is the result of the overlapping effects of multiple forces. The synergy factor *r* supports *C*, and the fine *β* supports $P_S$ and inhibits *D*. In addition, a very important influence is the three strategies’ cyclic dominance (as discussed in detail below). Due to r’s support for *C*, the $P_S$ cannot survive in high-cost areas. In the early stage of evolution, the increase of *C* provides the impetus for the evolution of *D*, and at the same time increases the exploitation of *P*, resulting in the punishment losing its effectiveness.

Compared with the *EI* punishment, the phase diagrams of the *EP* punishment seem straightforward, as displayed in Fig. 1c and d. When increasing the fine $\beta$ at a low value of cost $\alpha$ ($\alpha <0.3$ for $r=3.5$ and $\alpha <0.32$ for $r=3.8$), *P*_*B*_ gradually dominates the system to transition from the D (or $\mathrm C+D$) phase to the $\mathrm D+P_B$ phase, and finally forms the pure $\mathrm P_B$ phase. When the cost value is high, the $\mathrm P_B$ phase replaces the D (or $\mathrm C+D$) phase directly. It is worth noting that in the $\mathrm P_B$ phase, although the $P_B$ behaves in the same manner as the *C*, it is essentially different from the *C* because at this time $P_B$ has the punitive attribute; that is, once *D* invades $P_B$, it has the ability to eliminate it. In addition, *C* and $P_B$ coexist only when the cost is zero.

**Figure 2:**
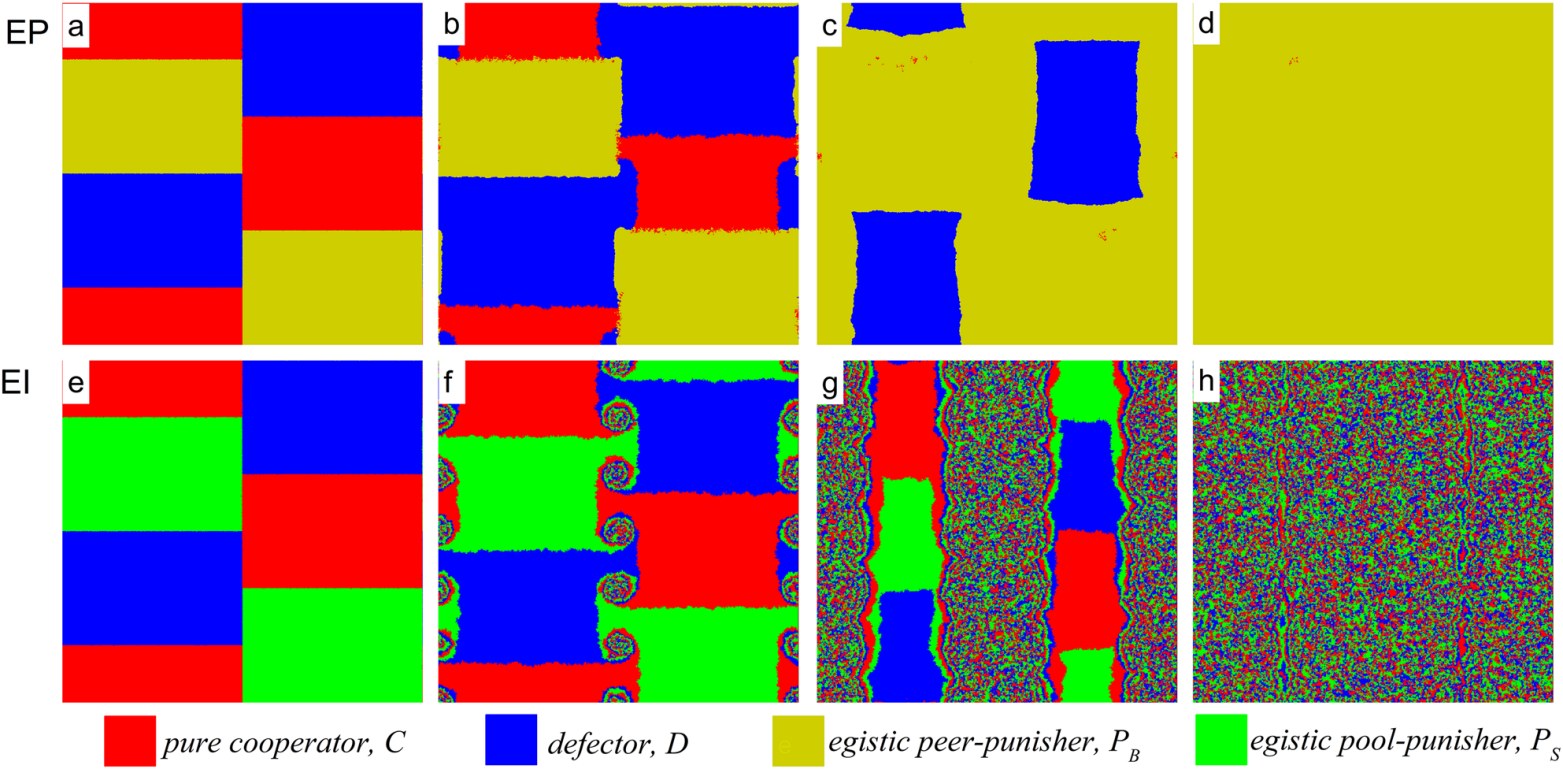
Evolution of three strategies under the *EI* and *EP* mechanisms. Snapshots from (**a**) to (**d**) are steps 1, 100, 1000, and 3000 under the *EP* mechanism, respectively, and snapshots from (**e**) to (**h**) are steps 1, 100, 300, and 500 under the *EI* mechanism, respectively. Results accrued for $r=2.0,\alpha =0.5,\beta =1.0$, and $L=1000$ with prepared initial distributions. The rock-paper-scissors phenomenon in the *EI* mechanism allows the egoistic pool punisher to coexist with the other two strategies. While in the *EP* mechanism, the defector first eliminates the pure cooperator and the egoistic peer punisher eventually eliminates the defector.

The presented fine-cost phase diagram shows clearly that $P_B$ has an absolute evolutionary advantage of eliminating *D*, while $P_S$ can survive at a lower-fine and higher-cost area. The three roles of *r*, $\beta$, and cycle dominance lead the disappearance of the evolutionary advantage of $P_S$ in high-cost and high-r areas. There are big differences in the phase transition types of the two punishment modes and the survival methods of two punishers due to the cycle-dominant role, which exists in the *EI* punishment mechanism but not in the *EP* punishment mechanism, as shown in Fig. 2.

Cyclic dominance, or multiple Nash equilibrium, is an important and common property in the system of three or more strategies. This phenomenon has been reported in previous studies, and we will not explain it in detail. Here, we are interested in why this phenomenon occurs in the *EI* but not in the *EP*, and what effect this dominant role has on the *EI* punishment mechanism.

In order to facilitate the observation of the competition between strategies, we show some typical snapshots of the strategies with prepared initial distribution under *EP* and *EI* mechanisms, as shown in Fig. 2. The results of the random initial distribution are shown in Figure S3 in the supplementary material. In the *EP* mechanism (Fig. 2a–d), the speed of the defector invading the cooperator is greater than that of the punisher invading the defector. Because the egoistic peer punisher ($P_B$) is the same as the pure cooperator if the group has no defectors, the boundary of these two cooperative strategies is not disturbed. Thus, leading the defector eliminates the cooperator first and then the punisher destroyed the defector cluster. After eliminating the defector, the egoistic peer punisher becomes a pure cooperator without paying any additional cost, and the group becomes a full-cooperative group with hidden punishment mechanisms. Once a mutated defector attacks the group, the punisher reappears to resist the defector’s invasion, which is driven by the force that protects their cooperation benefits. In the *EI* mechanism, the three strategies suppress each other, as shown in Fig. 2e–h. Swirls observed in the junction of the three strategies are rotating as well as extending gradually (Fig. 2f). In this rock-scissor-paper dynamic, defectors invade cooperators’ territory, and cooperators invade compensatory punishers while egoistic pool punishers defeat defectors in the border between them. The three strategies coexist when the evolution is stable.

**Figure 3:**
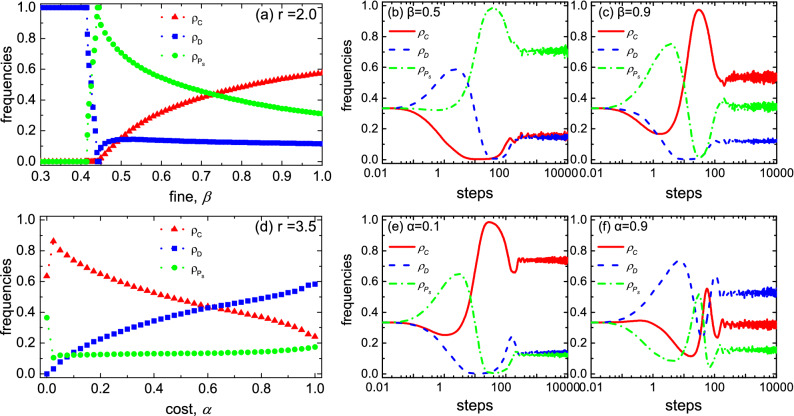
The effect of the punishment fine [graph (**a**)] and the cost [graph (**d**)] on the evolution of strategies in the *EI* mechanism. Graphs (**b**) and (**c**) show the evolution of strategies on a time scale when $r=2.0$; Graphs (**e**) and (**f**) show the evolution of strategies on a time scale when $r=3.5$. Results accrued for $\alpha =0.1$ in the upper layer, and for $\beta =0.5$ in the lower layer. Initially, the three strategies are randomly uniformly distributed. Results show $\alpha$ affects the level of cooperation and $\beta$ adjusts the proportion of the pure cooperator and the egoistic pool punisher in the coexistence phase of the three strategies.

Due to the strategies’ cyclic dominance, the influence of various parameters in the *EI* mechanism on cooperation is counter-intuitive. Figure 3 shows the influence of variables $\alpha$ and $\beta$ on the evolution of strategies.

When increasing the fine $\beta$ for a fixed value of cost $\alpha$ ($\alpha =0.1$) (Fig. 3a), the cooperation rate ($i.e.,\rho _C+\rho _{P_S}$ or $1-\rho _D$) was largely unchanged in the three strategies’ coexisting stage, although the proportions of the pure cooperator ($\rho _C$) and the punisher ($\rho _{P_S}$) changed. Moreover, the frequency of the punisher decreases in contrast to the intuition that the fine is good for the punisher but not for the defector, as explained by the evolutionary time scales. When the fine is small ($\beta =0.5$) (Fig. 3b), the damage to the defector and the reinforcement on the punisher are weak. The defector first occupies the evolutionary advantage when the cooperative synergy is low ($r=2.0$); then the punisher increases rapidly through the effect of strategies’ cyclic dominance. In contrast, when the fine is large ($\beta =0.9$) (Fig. 3c), the damage to the defector and the reinforcement of the punisher are strong. The punisher first increases rapidly, whereas the defector disappears. Under the influence of the cyclic dominance, the pure cooperator invades the punisher population and occupies the dominant position in a stable state. Therefore, the final result we see is that the fine’s main role is to adjust the proportion of the pure cooperator and egoistic pool punisher in the strategies’ coexistence phase, except to invade the pure defector group.

When increasing the cost $\alpha$ for a fixed value of fine $\beta$ ($\beta =0.5$) (Fig. 3d), the egoistic punisher’s frequency increases slightly, which contradicts common sense that the cost weakens the punisher. We compare the evolution of strategies on time scales under high- and low-cost conditions. When the cost is low ($\alpha =0.1$) (Fig. 3e), the punisher first dominates and weakens the defector. Through cyclic dominance, the pure cooperator increases rapidly in the population of punishers and dominates in the stable state. When the cost is high ($\alpha =0.9$) (Fig. 3f), first, the heavy cost greatly weakens the punisher, thus, the defector is dominant. Although the punisher has an opportunity to invade the defectors’ population, the pure cooperator destroys it and the punisher ultimately does not succeed. Therefore, adjusting the overall level of cooperation reflects the ultimate impact of cost by affecting the egoistic pool punisher’s initial status.

In addition, the non-intuitive effects of *r* in *EI* have also been explored in that the cooperation level decreases when *r* increases. Figure S4 in the supplementary material shows these results.

From the above results, we can see that the influence of parameters on the strategy occurs first in the *EI* mechanism, and then the cycle dominant role of the strategy appears, which makes the final role of the parameters change. It is worth mentioning that the effect of the cycle dominance in the three strategies’ coexistence state is only reflected in the first-order role; that is, if the punisher temporarily dominates under the influence of the parameters, the pure cooperator will ultimately dominate; if the defector has the advantage, the punisher dominates; and if the pure cooperator is dominant, the defector will ultimately prevail.

In general, egoistic punishment can effectively promote cooperation, whether through peer-punishment or pool-punishment methods. This result is robust to different strategy-update rules (See Figure S5 in the supplementary material for details). But the effects of promoting cooperation and the operating mechanisms behind them are very different.

### Comparison of egoistic and altruistic punishment

The above part fully explored egoistic punishment under the two punishment modes. In this part, we focus on the comparison between egoistic and altruistic punishment from the three following aspects: the level of cooperation when the evolution is stable, the type of EE, and the punisher’s survivability.

**Figure 4:**
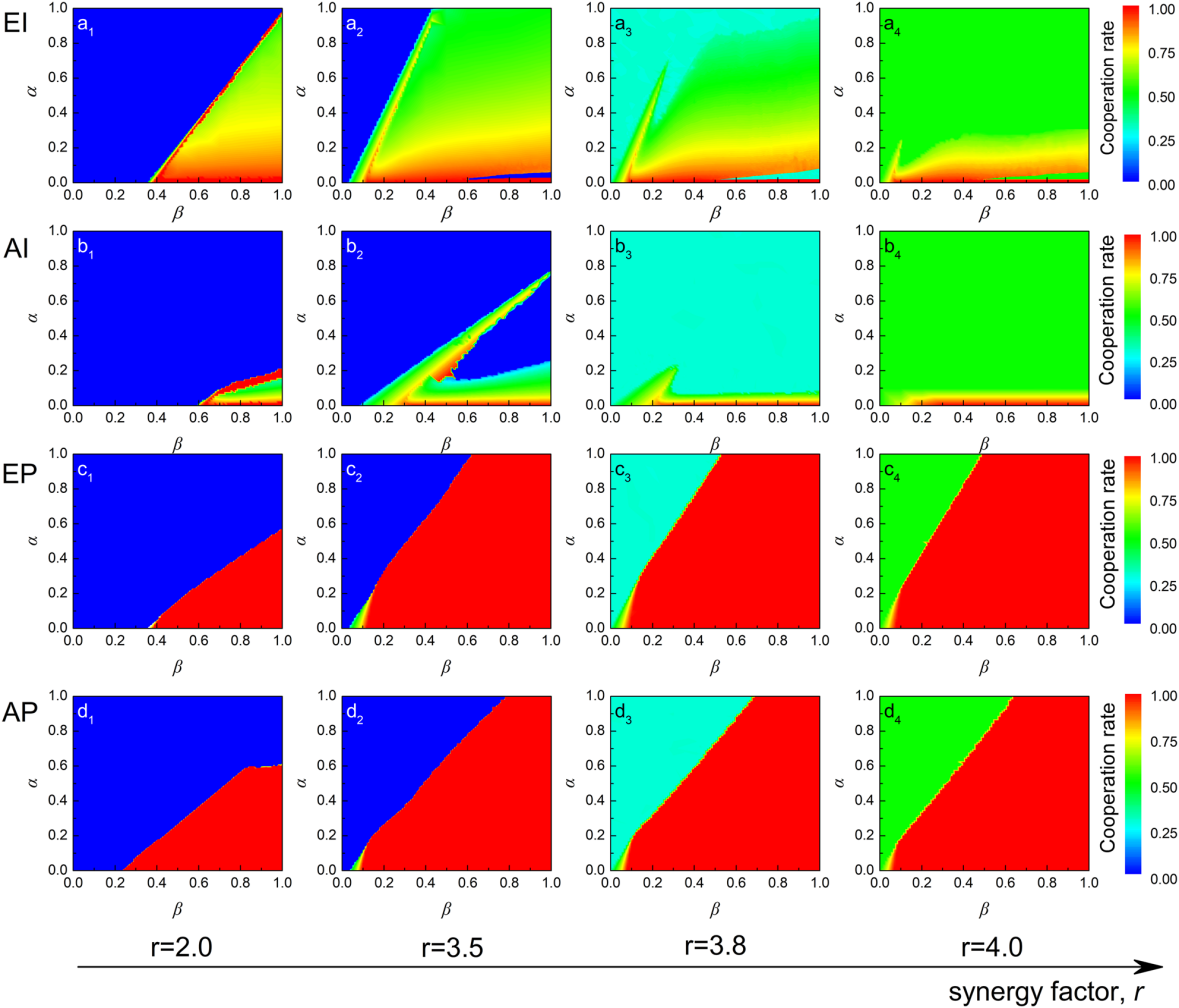
The levels of cooperation as a function of punishment cost ($\alpha$) and punishment fine ($\beta$) at $r = 2.0,3.5,3.8$,and 4.0 in four punishment mechanisms. From top to bottom, each row of graphs represents *EI*, *AI*, *EP*, and *AP* mechanisms, respectively. From left to right, each column represents $r = 2.0,3.5,3.8$, and 4.0. Results show egoistic punishment preforms better than altruistic punishment. *EP* can maintain a high level of cooperation, while *EI* can promote cooperation within a larger range of parameters for $r \le 3.5$.

First, we compared the level of cooperation. The cooperation rate is usually considered the ratio of cooperators (*C* and $P_i$, $i=R,G,S,or~ B$) in the population in an evolutionary stable state. To make the comparison more comprehensive, we studied the stationary ratio of cooperators when simultaneously changing the values of $\alpha$ and $\beta$ for different values of synergy factor *r*, as shown in Fig. 4. There is a significant difference between egoism and altruism in promoting cooperation. Under the peer-punishment mode (graphs ($c_i$) and ($d_i$)), cooperation rates of *EP* and *AP* can reach a large-scale, full cooperation level. However, egoistic punishment can promote cooperation with lower fines in high-cost areas when $r\ge 3.5$. Under the pool-punishment mode (graphs ($a_i$) and ($b_i$)), egoistic punishment promotes cooperation in a larger fine-cost area, especially in low-synergy-factor and high-cost conditions, compared with altruistic punishment. Despite a small transition interval (as Fig. 1 explains), the role of egoistic punishment has a very significant advantage. In summary, egoistic punishment preforms better than altruistic punishment in promoting cooperation under the two punishment modes. Moreover, *EP* can maintain a high level of cooperation, while *EI* can promote cooperation within a larger range of parameters when $r\le 3.5$.

The advantages of egoistic punishment seem to disappear in the condition that $r=2.0$ under peer-punishment, as Fig. 4 ($c_1$) and ($d_1$) show. In fact, the defector in altruistic punishment is punished much more severely than in egoistic punishment, although the egoistic punishers’ fitness is larger than that of altruistic punishers. In detail, if we suppose the number of punishers in the group is the same, a defector loses benefits $\beta *N_{P_R}$ when encountering altruistic punishers ($P_R$), whereas the defector loses benefits $\beta *r/5*N_{P_B}$ when encountering egoistic punishers ($P_B$). If we make the losses for defectors in both games the same, setting $\beta(P_R)=\beta(P_B)*r/5$, clearly, egoistic punishment will reach a steady state of full cooperation and evolve faster than altruistic punishment. This result shows that if defectors are punished to the same degree, obviously, *EP* promotes cooperation more efficiently than *AP* by increasing punishers’ fitness.

Promoting cooperation under both motives significantly depends on the punishment cost ($\alpha$) and fines ($\beta$). The harsher the punishment, the lower the cost and the greater the possibility of promoting cooperation, which is also the requirement of altruistic punishment to promote cooperation. Egoistic punishment can promote cooperation in areas with lower fines and higher costs, reflecting the notion that egoistic punishment has a more tolerant requirement for punishment conditions. This result provides another way to improve cooperation through punishment; that is, a punishment mechanism that compensates the punisher and tolerates the defector. On one hand, compensation covers part of the punisher’s cost to improve its fitness; on the other hand, tolerant punishment can also weaken the defector’s fitness to promote cooperation. Results show that the latter way—where egoistic punishment goes–performs better.

**Figure 5:**
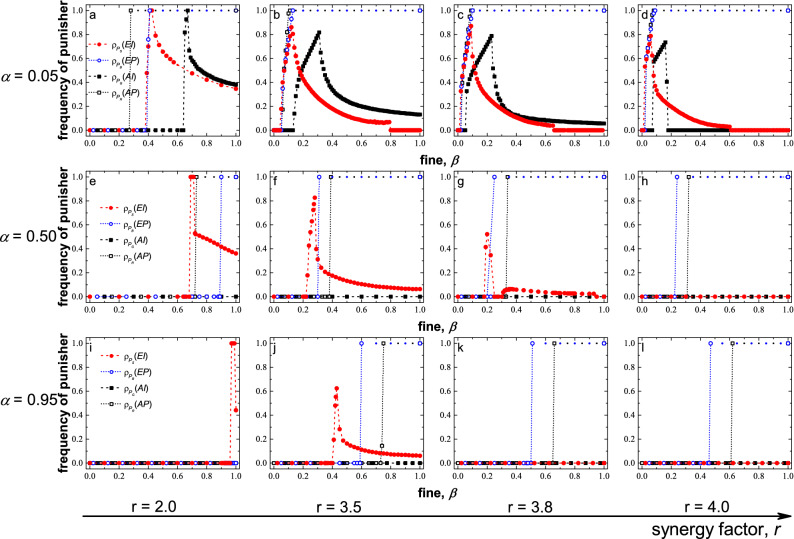
The frequencies of punishers as a function of punishment fines in four punishment mechanisms at different costs ($\alpha$) and synergy factors (*r*). Red solid circles represent egoistic pool punisher ($P_S$) and blue circles mean egoistic peer punisher ($P_B$). Black solid squares represent altruistic pool punisher ($P_G$) and black hollow squares mean altruistic peer punisher ($P_R$). Results show survivability of $P_S$ and $P_G$ are stronger than that of $P_B$ and $P_R$, but adaptability is worse. Moreover, $P_S$ can survive better in lower-fine areas than $P_G$. After the peer punishers occupy the population, they behave like pure cooperators and are easily invaded by pure cooperators, so they cannot be identified when there is no defector. Therefore, we marked the first and last time the beta value of the population occupied by the peer punishers in the graphs, while ignoring the intermediate value for easy observation.

Then, we compared the four types of punishers’ survivability under the conditions that the punishment cost is low ($\alpha = 0.05$), medium ($\alpha = 0.5$), and high ($\alpha = 0.95$), as shown in Fig. 5. When increasing the fine $\beta$ at a low cost value in the peer-punishment mode, altruistic punishers ($P_R$) first prevail and occupy the population, as followed by the egoistic punishers ($P_B$). After the peer punishers occupy the population, they behave like pure cooperators and are easily invaded by pure cooperators, so they cannot be identified when there is no defector. Therefore, we marked the first and last time the $\beta$ value of the population was occupied by the peer punishers in the graphs, while ignoring the intermediate value for easy observation. As the cost increases, it becomes increasingly difficult for the peer punishers to survive. When increasing the *r*, $P_B$ is more survivable than $P_R$.

**Figure 6:**
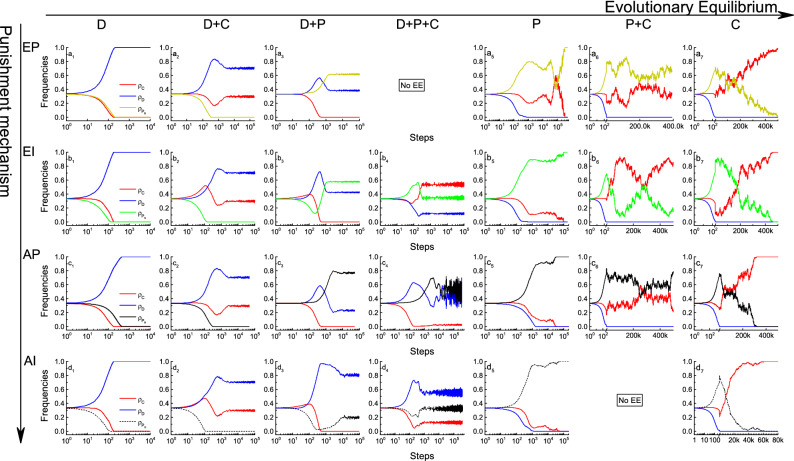
Evolutionary equilibrium states of four punishment mechanisms. We show the evolutionary equilibrium state of the strategies from full defective (*D*) to full cooperation (*C*). The red line indicates pure cooperator, the blue line indicates the defector, the dark yellow line in *EP* indicates the egoistic peer punisher ($P_B$), the green line in *EI* is the egoistic pool punisher ($P_S$), the black dash line in *AP* is the altruistic peer punisher ($P_R$) and the black solid line in *AI* is the altruistic pool punisher ($P_G$). “No EE” stands for no such evolutionary equilibrium state under the current punishment mechanism; that is, these strategies cannot coexist. The strategy evolution under the four mechanisms is different; thus, we try to observe the evolutionary stable state under the same parameter combination ($r, \alpha , \beta$) in the same EE state. D phases are obtained for (2, 0.5, 0.5); D+C phases for (3.8, 0.8, 0.2); D+P phases for (3.5, 0.1, 0.2) in *AI* and (3.5, 0.1, 0.1) for other mechanisms; D+C+P phases for (2, 0.1, 0.9) in *AI* and *EI*, and (2, 0.6, 0.9) in *AP*. The full-cooperation phases accrued for $r = 4$. In *AI*, P phase is obtained for (*r*, 0, 0.3) and C phase is obtained for (*r*, 0.0001, 0.3). In *EI*, P phase is obtained for (*r*, 0, 0.2), C phase is obtained for (*r*, 0.0001, 0.2), and P+C phase is obtained for (*r*, 0, 0.6). In fact, when $\alpha = 0$ punishers are the same as cooperators. In *EP* and *AP*, although cooperators and punishers cannot be distinguished in theory, simulation results show that all three states of C, P, and C+P can be observed in the full-cooperation area.

In the pool-punishment mode, when increasing the fine $\beta$ at a low cost value, the pool punishers ($P_S$ and $P_G$) rise rapidly and then decrease gradually as the punishment fine transitions from tolerant to severe. The egoistic punishers ($P_S$) first prevail but the altruistic punisher’s ($P_G$) frequency is higher than $P_S$. As the cost increases, $P_G$ has no survivability, while $P_S$ can survive at a high-fine area. As *r* increases, the $P_S$ can survive with a lower fine.

Peer punishers have strong survivability within a considerable range of parameters, but are easily invaded by pure cooperators, while the pool punishers’ survival parameters range is quite limited. Compared to motivation, egoistic punishers can survive with higher costs and lower fines than altruistic punishers.

Finally,we analyzed the EE of egoism and altruism in two punishment modes to compare the dynamic-evolution process shown in Fig. 6 (the results can also be obtained by random initial distribution). EE is the ultimate stable proportion to which an evolutionarily changing population converges, referring to Maynard Smith’s original definition. Therefore, a game of three strategies has no more than seven EE states. Analyzing the EE that emerged in four punishment mechanisms shows three regions from high-cost lenient punishment to low-cost severe punishment. First, in the defection prosperous area, cooperation emerges and forms a coexistence of defectors and cooperators, and finally cooperation completely occupies the population. The factors influencing the evolution of cooperation in different regions are also different. In the defection prosperous area, two types of EE states exist: D and C+D. The D phase appears when $r=2.0$ and 3.5 (the blue region in Fig. 4), and the C+D phase emerges when $r=3.8$ and 4.0 (the cyan and green region in Fig. 4), which reveals that synergy factor *r* supports cooperation. In the transitional area, two equilibrium states—D+$\mathrm P_i$ and $\mathrm (C+D+P_i)_c$($i=R,S$ and *G*)—emerge. In the *EP* mechanism, only the $\mathrm D+P_B$ phase exists because the pure cooperator cannot take a free ride from the *EP*. This stage reflects the influence of punishment in promoting cooperation. Interestingly, the full-cooperation area has three EE states: P, P+C, and C, but they do not appear in all four punishment models. The P+C phase cannot be observed in the *AI* mechanism, which reflects the mandatory nature of the institutional punishment the government implement. In peer punishment (*EP* and *AP*), observing all three EEs is understandable because the individual punisher becomes the same as the pure cooperator in the absence of defectors. This stage reflects the influence of cyclic dominance in the three strategies of EE.

## Discussion

The evolution of costly punishment is a puzzle due to the existence of pure cooperators’ second-order free-riding. In this study, we try to explain this confusion from the perspective of punishment motivation; that is, we aim to answer why the punisher is willing to pay extra punishment costs. Inspired by theory and human nature in reality, we proposed the punishment of egoism. Results indicate egoistic punishment can evolve and effectively promote cooperation within a large parameter range, whether in a well-mixed or structured population, or through peer-punishment or pool-punishment modes. This result is also robust to different strategy-updating rules. The main difference is egoistic peer-punishment can quickly eliminate the defectors and evolve the population into full cooperation, while egoistic pool-punishment can evolve and maintain a certain level of cooperation in lower-fine and higher-cost areas. Compared with altruistic punishment, egoistic punishment can promote cooperation in a lower-fine and higher-cost area, especially in the pool-punishment mode, and the egoistic punishers have stronger survivability.

Previous studies explained the evolution of costly punishment in many ways, as reviewed in the introduction. This study supplements existing solutions from the perspective of motivation. This study has similarities with existing solutions in terms of expression, such as when the egoistic punisher retrieves part of the payoffs from the defectors, and this behavior partially compensates for the punishment cost. It seems to be consistent with the solution to reduce punishment costs. On the other hand, the punishers gain payoff by punishing the defectors, while the cooperators do not increase payoffs for failing to punish the defectors. Therefore, egoistic punishment avoids second-order free-riding to a certain extent. Despite these similarities, the egoist motive driving these behaviors is the innovation of this article. The egoistic punishment here is not to retaliate or vent emotions, but to protect the deserved benefits of cooperation. This motive is not uncommon, and it can be seen in each of us as ordinary people. Moreover, the egoistic punishment in this study is quite tolerant; that is, the punisher only requires part of the payoff for the defector obtained from the punisher’s cooperation, not all or even more. Experiments show that egoistic punishment is an intuitive and effective mechanism. Egoistic punishment can not only explain the emergence and evolution of costly punishment, but also effectively promote cooperation.

This research also offers novel and meaningful enlightenment for solving the problem of CPR. For example, during fishing prohibition period, if the cooperator found a fisher who violated the order, he could request that the finder own part of the caught fish for punishment, and the punisher would also pay the cost of supervision. Similarly, there are public transportation cases; for example, if a driver (or pedestrian) finds another driver violating traffic rules, which may cause public traffic jams, he can punish the driver by taking photos to report the incident and get rewards (or compensation). The egoistic punishment is more easily stimulated compared with the altruistic punishment. Under the egoistic pool-punishment, the punishment cost is the key factor to control the level of cooperation in population, and the fine coefficient is not as high as possible. A moderate fine coefficient can ensure the sustainability of punishment. The altruistic pool-punishment is only applicable when the cost is low. This result implies that if the third-party enforcement cost is higher, such as a corrupted law-enforcement system, the egoistic punishment will prevail. There is no need to consider the abnormal influence of parameters in the peer-punishment mechanism, which is an intelligent system with hidden punishment functions driven by egoism or altruism, but the applicable parameter range of egoistic punishment is wider.

Egoistic punishment is a fair punishment. The punisher is protecting its own cooperative benefit. This kind of individual behavior promotes collective cooperation. The findings mirror the evidence in human history that homo economicus not only builds up the market’s foundation, but also the order of justice and fairness.

This study establishes a model of the egoistic punishment mechanism, which is simple, but captures the main factors. In future studies, one can continue to add more realistic factors to perfect this series of work, such as the randomness of punishment and the existence of anti-social punishment.

## Methods

Our model is based on the spatial PGG and entails the cooperator (*C*), defector (*D*) and punisher ($P_i,(i=B,S,R,G)$). Our experiment contained two stages in each round game: the PGG stage and the punishment stage. In detail, before the PGG stage, institutional punishers $P_G$ and $P_S$ pay cost $\alpha$ to build the public punishment pool. In the PGG stage, the two cooperative strategies C and $P_i,(i=B,S,R,G)$ contribute *c* to the public good while defectors contribute nothing. We multiplied the sum of all contributions by the synergy factor *r* ($r>1$), equally shared among interacting individuals, irrespective of their strategies. The factor *r* reflects the synergetic effects of cooperation and determines the value of public goods. After the PGG, peer punishers $P_B$ and $P_R$ decide whether to pay costs $\alpha$ to punish or not, according to whether the group has defectors. In the punishment stage, if the group has punishers $P_G$ and $P_R$, each defector will bear fine $\beta$ and $P_i,(i = G,R)$ will not receive rewards; if the group has punishers $P_S$ and $P_B$, each $P_i,(i= B,S)$ will retrieve $\beta *r/g$ from a defector’s payoff as compensation. Note that the payoff *r*/*g* of a defector comes from the punisher’s cooperation. Hence, the total fine for a defector relates to the number of punishers in peer punishment, which means if the group has no punishers, the defector will not be punished, and their payoffs will not be reduced.

The spatial game experiment is carried out on the four-neighbor lattice with periodic boundaries. Players are arranged into overlapping five-person groups such that each player at site *x* serves as a focal player in the group, formed with its four nearest neighbors. Accordingly, each individual belongs to *g*($g = 5$) different groups. To obtain an outcome, all players play five elementary PGGs by following the same strategy in every group with which they are affiliated. In an elementary five-person PGG, each player has a random strategy: *C*, *D*, or $P_i,(i=B,S,R,G)$. Denoting the number of *C*, *D*, and $P_i$ in a given group G by $N_C, N_D$, and $N_{P_i}$, respectively, yields $N_C+N_D+N_{P_i}=5$ in each group. The payoff equations are as follows:

1 $$\begin{aligned} P_C=\sum _{g=1}^{5}[(N_C+N_{P_i})*r/5-1] (i=B,S,R,G) \end{aligned}$$

The pure cooperator’s payoff is the same in four punishment forms. However, defectors’ and punishers’ payoffs are different among four types of punishments. In *EP* punishment,

2 $$\begin{aligned} P_D= & {} \sum _{g=1}^{5}[(N_C+N_{P_B})*r/5-N_{P_B}*\beta *r/5] \end{aligned}$$

3 $$\begin{aligned} P_{P_B}= & {} \sum _{g=1}^{5}[(N_C+N_{P_B})*r/5-1-\alpha *N_D+\beta *r/5*N_D] \end{aligned}$$

In *EI* punishment^52^,

4 $$\begin{aligned} P_D= & {} \sum _{g=1}^{5}[(N_C+N_{P_S})*r/5-N_{P_S}*\beta *r/5] \end{aligned}$$

5 $$\begin{aligned} P_{P_S}= & {} \sum _{g=1}^{5}[(N_C+N_{P_S})*r/5-1-\alpha +\beta *r/5*N_D] \end{aligned}$$

In *AP* punishment, we refer to the setting of reference^14^

6 $$\begin{aligned} P_D= & {} \sum _{g=1}^{5}[(N_C+N_{P_R})*r/5-\beta *N_{P_R}] \end{aligned}$$

7 $$\begin{aligned} P_{P_R}= & {} \sum _{g=1}^{5}[(N_C+N_{P_R})*r/5-1-\alpha *N_D] \end{aligned}$$

In *AI* punishment, we refer to the setting of reference^13, 14^

8 $$\begin{aligned} P_D= & {} \sum _{g=1}^{5}[(N_C+N_{P_G})*r/5-\beta *f], where ~ if ~ N_{P_G}=0, f=0 ; else ~ f=1. \end{aligned}$$

9 $$\begin{aligned} P_{P_G}= & {} \sum _{g=1}^{5}[(N_C+N_{P_G})*r/5-1-\alpha ] \end{aligned}$$

Agents update strategies according to the Fermi updating rule (FUR) in each round of the game to be cooperators, defectors, and punishers. They perform the strategy update through stochastic imitation of more successful neighbors, determined by the Monte Carlo step (MCs). First, a randomly selected player *x* plays the PGG with its four interactive partners and obtains a payoff from all *g* groups to which it belongs. Thus, the overall payoff is $P_x=\sum _{g}P_{x}^{g}$. Next, one of the four nearest neighbors of player *x* is chosen at random, called co-player *y*, and *y* also acquires its payoff $P_y$ in the same way. Finally, player *x* imitates the strategy of player *y* with the following probability:

10 $$\begin{aligned} q=\frac{1}{{1+exp[P_x-P_y]/K}} \end{aligned}$$

where *K* quantifies the level of uncertainty of strategy adoptions. In the limiting case, $K\rightarrow 0$, player *x* copies the strategy of player *y* if and only if $P_x<P_y$. For $K\rightarrow \infty$, underperforming strategies may also be sometimes adopted. Without loss of generality, we set $K = 0.5$, implying that better-performing strategies are readily imitated, but it is not impossible to adopt the strategy of a player performing worse. Each MCs gives a chance for players to learn a fruitful strategy from one of their neighbors, on average.

It should be pointed out that, in addition to the FUR as described above, we also use other updating rules to verify egoistic punishment’s effectiveness. First, we use the Myopic best-response rule (MBR)^53^, where player *x* imitates the strategy ($x^{'}$) that is randomly select from the remaining two strategies with the above probability in Eq. (10) where $P_{x^{'}}$ replaces $P_y$. We also consider the best-take-over update rule (BUR)^54^, where player *x* updates its strategy of a deterministic selected from the neighbor’s strategy with the highest payoff. In addition, we also condiser the aspiration-driven update rule (AUR)^55^, where player *x* updates it’s strategy dependent on the difference between the current payoff and the aspirational payoff $P_{xa}$ which is defined as $P_{xa}=k_{x}A$, where $k_{x}$ is the connectivity of player *x* ($k_{x}=4$ for the square lattice) and *A* is the aspirational level (*A*=0.3 in the experiment). AUR introduces the impact of strategic mutations.

To get accurate computational results, the final frequencies $\rho _j$, of $j=C,D,P_i(i=B,S,R,G)$, on the square lattice with $L^2$ sites, are determined by averaging over a sampling time ($t=5000$) after a sufficiently long relaxation time ($10^6$ iterations). *L* is chosen from 400 to 4000 for the simulation results unless otherwise specified. The initial strategy’s random distribution may foster a strategy’s sudden disappearance. We have two methods to solve this problem: one method aims to increase the population size, the other strives to use a prepared initial distribution. We chose the latter method to save experiment time, but also used the former to test.

## Supplementary Information

Supplementary Information
