# Supplementary material for: Egoistic punishment outcompetes altruistic punishment in the spatial public goods game
Source: Sci Rep. 2021 Mar 22;11:6584. doi: 10.1038/s41598-021-85814-1 (PMC7985383; doi:10.1038/s41598-021-85814-1)
Supplement: Supplementary file 1 — Supplementary Information [file 41598_2021_85814_MOESM1_ESM.pdf]

Supporting Materials:

Egoistic Punishment Outcompetes Altruistic Punishment in the Spatial Public Goods Game

Juan Li, Yi Liu, Zhen Wang, Haoxiang Xia

1. Analysis of egoistic punishment in a well-mixed population

In the public goods game with egoistic punishment in a well-mixed population, there are three strategies in the population: (a) the pure cooperators (*C*) contribute their effort, (b) the defectors (*D*) contribute nothing but share the fruits of cooperation, and (c) the punishers not only contribute their effort, but also pay a cost α to acquire a reward β from the fine imposed on defectors. Therefore, the behaviors of punishers in two punishment modes are different. In peer punishment (EP), the punisher (P_B) pays a cost before the PGG stage, while in pool punishment (EI), the punisher (P_S) pays the cost after the PGG stage and finds there is defectors in the group. Assuming that the population is the group, the payoffs for different strategies *C, D* and $P_i(i = B, S)$ are then determined by the relative frequencies x, y and $z_i(i = B\ or\ S)$ of the three strategies, and x+y+$z_i = 1$.

The replicator equation of the evolution of the frequencies of the strategies is given by

$$\dot{x}_i = x_i(f_i - \bar{f}) \ , \tag{1}$$

where $\bar{f} = xf_C + yf_D + z_i f_{P_i}$ is the average payoff in the population. For simplicity and without loss of generality, we set the contribution of cooperation equal to 1. The payoffs for cooperators, defectors and punishers are then given by

$$\begin{aligned} f_C &= r(x + z_i) - 1 \\ f_D &= r(x + z_i) - r\beta * z_i \\ f_{P_B} &= r(x + z_B) - 1 - \alpha + r\beta * y \\ f_{P_S} &= r(x + z_S) - 1 - \alpha * y + r\beta * y \end{aligned} \tag{2}$$

Next is the analysis of the replicator dynamics. First of all, we solve the equation with the initial state of different types of strategies under fixed parameters of $r, \alpha, and\ \beta$. Then we solve the equation when varying the fine and the cost at different value of synergy factor r. The transition points and the type of phase transitions are identified from the data collected obtained by Matlab. Finally, the phase boundaries, separating different solutions, are plotted in the full fine-cost phase diagrams shown in Figure S1 and S2.

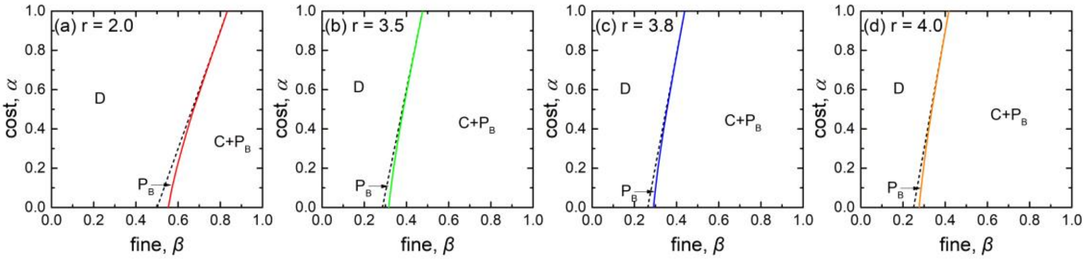

Figure S1. The fine-cost phase diagrams of egoistic peer-punishment in the well-mixed population. There are three solutions of the pure D, the pure P_B, and the coexist of C+P_B. Peer egoistic punishers replace defectors and then are invaded by pure cooperators as β

increases or as α decreases. The boundaries move left as *r* increases.

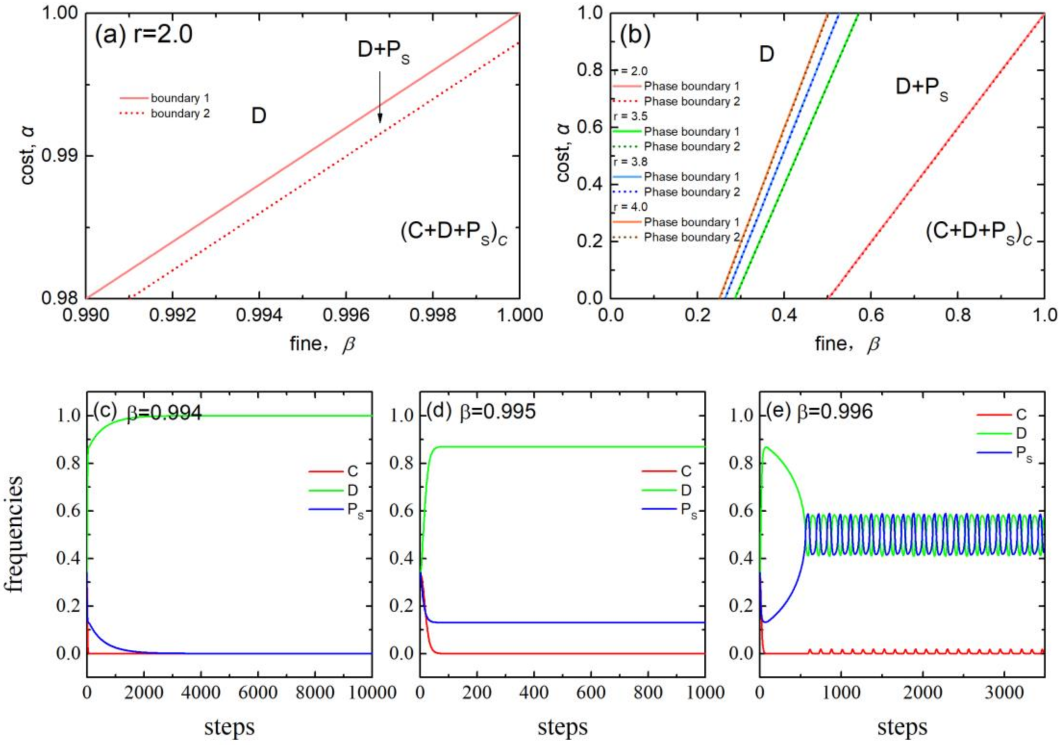

Figure S2. The fine-cost phase diagrams of egoistic pool-punishment in the well-mixed population. There are three solutions of the pure D, coexist of D+P_S, and the cycle dominance of C+D+P_S. Graph (a) is the enlarged of graph (b) for r=2.0. One can observe a consecutive transition from the pure D phase to the D+P_S phase, then to the C+D+Ps phase. Graph (c)-(e) show the evolution process of three strategies under three phase transitions shown in graph (a) when $\alpha = 0.99$. Graph (b) fully shows the phase transitions at different value of *r*. the D+P_S phase is very short, so the two boundaries almost coincide.

2. Cycle dominance phenomenon with random uniform initial distribution

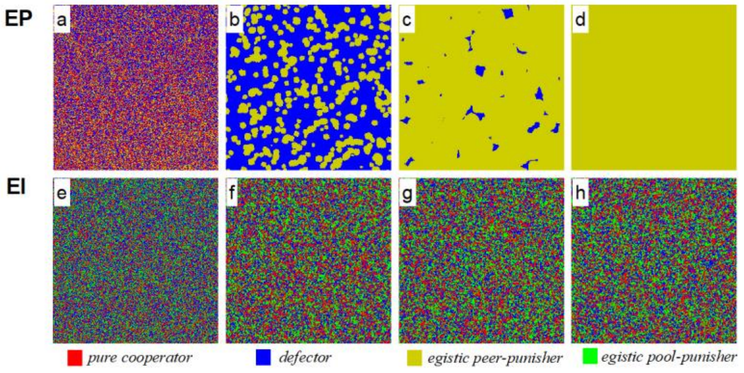

Figure S3. The evolution of three strategies under EP and EI punishment mechanism. Snapshots from (a) to (d) are steps 1, 200, 500, and 1000 under the EP mechanism respectively, and snapshots from (e) to (h) are steps 1, 100, 300, and 500 under the EI

mechanism respectively. Results accrued for $r = 2.0$, $\alpha = 0.5, \beta = 1.0$, and $L = 1000$ with random uniform initial distributions. In this case, the system of EP evolves into the pure punisher state, while the three strategies coexist in EI system. In fact, there is a three-strategy cycle dominant phenomenon in the EI system, but we cannot observe it from the random initial distribution

3. The non-intuitive effect of synergy factor

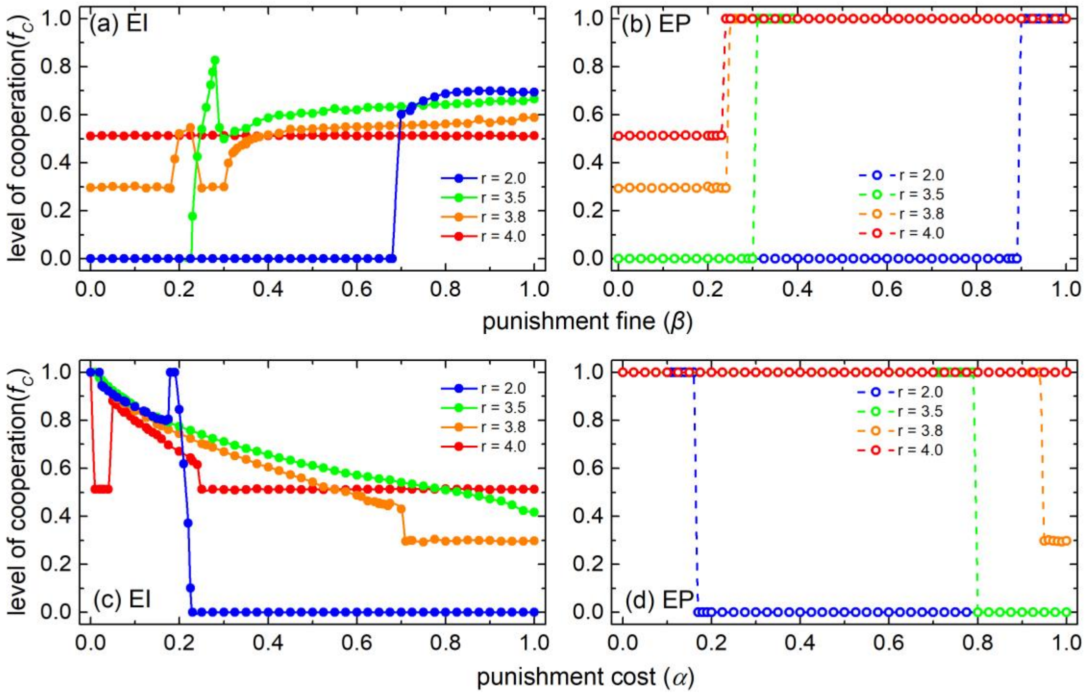

Figure S4. The frequencies of cooperation (f_C) as the function of the punishment fine (β) and the punishment cost (α) at different *r*. (a) and (c) are the results of egoistic pool-punishment, represented by the solid circles; (b) and (d) are the results of egoistic peer-punishment, indicated by the hollow circles. Colors from blue, green, orange to red represent the value of *r* from 2.0, 3.5, 3.8 to 4.0, respectively. Results of (a) and (b) accrued for $\alpha = 0.5$; (c) and (d) were obtained for $\beta = 0.5$. In EI, the advantage of the punisher decreases as costs increase. The punisher has an evolutionary advantage at very low cost, but it is replaced by pure cooperator since the cycle dominance, we have not observed the effect of punishment for r = 4.0 in (c). As α increases, From the three-strategy coexistence to a temporary full-punishers state, and then enter the state of competition between the punisher and the defector, until the cost burden makes the punisher unable to survive. So, there is a sudden change of r = 2.0 in graph (c). The performance of EP in promoting cooperation is better than that of EI under the same parameters. The situation is more complicated in EI, and the impact of r on cooperation is not intuitive.

Due to the cyclic dominance of strategies, some counterintuitive phenomena emerge in the egoistic pool-punishment. Figure S4 shows that the effects of r on the evolution of cooperation under the EP and EI mechanisms differ. In the EP mechanism, as *r* increases, the level of cooperation increases, consistent with previous studies, because r supports

cooperation. In contrast, in the EI mechanism, as *r* increases, the level of cooperation gradually decreases. This phenomenon can be clearly observed in $\beta > 0.7$ of graph (a) or $\alpha < 0.2$ of graph (c) due to the cyclic dominance of three strategies. The larger the r, the more advantageous the cooperator. Once cooperators become the dominant players, defectors will have more opportunities for exploitation, thereby reducing the overall level of cooperation.

4. The robust of strategy updating rules

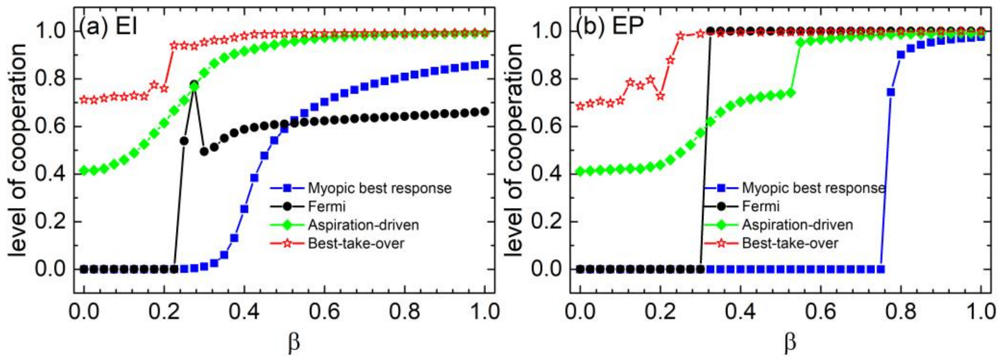

Figure S5. Frequency of cooperation in EI and EP mechanisms under different strategy updating rules. Results are obtained for r=3.5 and $\alpha = 0.5$. Cooperation refers to the proportion of cooperators and punishers in the population after evolution is stable. It turns out that the egoistic punishment can effectively promote cooperation under four different update rules. In EI punishment, Best-take-over (BUR) and Aspiration-driven (AUR) rules are the top two performers. Fermi (FUR) and Myopic best response (MBR) cannot promote cooperation under low fines ($\beta \leq 0.21$). In the middle fine area ($0.21 < \beta \leq 0.5$), FUR performs better, while in the high fine area, MBR performs better. In EP punishment, BUR is still the best performer, then is FUR when $\beta > 0.3$. Below this value of fine, the AUR performs better. In short, the FUR is neither the best nor the worst in promoting cooperation, but it expresses the bounded rationality. Therefore, we used this rule in the spatial experiments of this article. For a detailed description of each update rule, see the Method section in the main text.
